# Pre-assessment and management of long COVID patients requiring elective surgery: challenges and guidance

**DOI:** 10.1186/s13741-023-00305-3

**Published:** 2023-06-05

**Authors:** Sophie Boles, Sundar Raj Ashok

**Affiliations:** Department of Anaesthesia and Intensive Care Medicine, Croydon University Hospital, Surrey, UK

**Keywords:** COVID-19, Functional capacity, Guidelines, Long COVID, Preoperative, Assessment, Perioperative, Postoperative

## Abstract

Whilst most patients infected with COVID-19 make a full recovery, around 1 in 33 patients in the UK report ongoing symptoms post-infection, termed ‘long COVID’. Studies have demonstrated that infection with early COVID-19 variants increases postoperative mortality and pulmonary complications for around 7 weeks after acute infection. Furthermore, this increased risk persists for those with ongoing symptoms beyond 7 weeks. Patients with long COVID may therefore also be at increased postoperative risk, and despite the significant prevalence of long COVID, there are minimal guidelines on how best to assess and manage these patients perioperatively. Long COVID shares several clinical and pathophysiological similarities with conditions such as myalgic encephalitis/chronic fatigue syndrome and postural tachycardia syndrome; however, there are no current guidelines for the preoperative management of these patients to help develop something similar for long COVID patients. Developing guidelines for long COVID patients is further complicated by its heterogenous presentation and pathology. These patients can have persistent abnormalities on pulmonary function tests and echocardiography 3 months after acute infection, correlating with a reduced functional capacity. Conversely, some long COVID patients can continue to experience symptoms of dyspnoea and fatigue despite normal pulmonary function tests and echocardiography, yet demonstrating significantly reduced aerobic capacity on cardiopulmonary exercise testing even a year after initial infection. How to comprehensively risk assess these patients is therefore challenging. Existing preoperative guidelines for elective patients with recent COVID-19 generally focus on the timing of surgery and recommendations for pre-assessment if surgery is required before this time interval has elapsed. How long to delay surgery in those with ongoing symptoms and how to manage them perioperatively are less clear. We suggest that multidisciplinary decision-making is required for these patients, using a systems-based approach to guide discussion with specialists and the need for further preoperative investigations. However, without a better understanding of the postoperative risks for long COVID patients, it is difficult to obtain a multidisciplinary consensus and obtain informed patient consent. Prospective studies of long COVID patients undergoing elective surgery are urgently required to help quantify their postoperative risk and develop comprehensive perioperative guidelines for this complex patient group.

## Introduction

The COVID-19 pandemic has resulted in an unprecedented strain on healthcare worldwide. Acute infection with early SARS-CoV-2 variants increases postoperative mortality and pulmonary complications, and indeed, this increased risk persists until around 7 weeks after the acute infection (COVIDSurg Collaborative, GlobalSurg Collaborative 2021). Additionally, those with persisting symptoms beyond 7 weeks have a significantly higher postoperative mortality rate than those whose symptoms have resolved (COVIDSurg Collaborative, GlobalSurg Collaborative 2021). Around 1 in 33 people in the UK report persistent symptoms following COVID-19 infection, termed ‘long COVID’ (Office For National Statistics 2023). Despite the significant prevalence and the potentially increased postoperative risk for those with persistent symptoms, there are currently limited guidelines on how best to pre-assess and manage these patients when they require surgery. This is complicated by the varied presentation of long COVID and inconsistencies in defining it. Whilst the UK Office of National Statistics defines long COVID as COVID-19 symptoms that have persisted for more than 4 weeks, the National Institute for Health and Care Excellence (NICE) describes the post-COVID-19 syndrome as symptoms continuing for more than 12 weeks, and there are multiple other time frames used internationally (Office For National Statistics 2023; National Institute for Health and Care Excellence 2021). Furthermore, although two of the most common symptoms include dyspnoea and fatigue, there are many others reported (Office For National Statistics 2023). Most, if not all, are non-specific. Therefore, developing guidelines for recognising, assessing and optimising these patients preoperatively is challenging.

## Long COVID and chronic fatigue syndrome

Parallels between long COVID and myalgic encephalitis/chronic fatigue syndrome (ME/CFS) and postural tachycardia syndrome (POTS) have been made in the literature (Lopez-Leon et al. 2021). Fatigue is the most frequently reported symptom in those with long COVID (Office For National Statistics 2023), which can be severe and debilitating, and the flares described are arguably similar to the relapses seen in ME/CFS. Unfortunately, there are no specific guidelines for the preoperative management of ME/CFS sufferers to help aid the development of something similar for long COVID. Indeed, whilst exercise programmes before major surgery can be beneficial in reducing postoperative morbidity (Wynter-Blyth and Moorthy 2017), NICE no longer recommends graded exercise therapy to patients with ME/CFS due to the minimal evidence supporting an improvement in symptoms, and in fact, many patients with ME/CFS report that it exacerbates their condition (National Institute for Health and Care Excellence 2021). Long COVID patients may pose similar complexities in terms of optimising their functional capacity before surgery. Long COVID patients also report symptoms of autonomic dysfunction as seen in POTS sufferers; again, perioperative guidelines for the latter are limited, but suggestions include preoperative intravenous hydration and the continuation of beneficial medications, such as beta blockers (Ruzieh et al. 2018).

## Pathophysiology of long COVID

The pathophysiology of long COVID is a subject of ongoing research, and several hypotheses have been proposed. Given the diverse and multi-system presentation of long COVID, it is likely that the underlying process is complex and multimodal in nature. One theory attributes the symptomatology to the ongoing elevation of pro-inflammatory cytokines; levels of interleukin-1β, interleukin-6, tumour necrosis factor α (Schultheiß et al. 2022) and interferons (Phetsouphanh et al. 2022) correlate positively with long COVID symptoms 8 months after initial infection. Interestingly, the persistence of proteins from SARS-CoV-2 in long COVID sufferers has also been noted (Swank et al. 2022); perhaps this ongoing viral presence drives a chronic proinflammatory process in long COVID. Intriguingly, just as clinical parallels between long COVID and ME/CFS have been described, there is evidence that these diseases may also share common underlying mechanisms. Immune dysregulation may lead to the reactivation of certain viruses, such as the Epstein-Barr virus (Peluso et al. 2023); there is also some evidence of viral involvement in the development of ME/CFS (Ariza 2021). A persistently altered gut microbiome has been noted in long COVID patients (Liu et al. 2022), and gut dysbiosis is also described in ME/CFS (König et al. 2021). Additionally, evidence of endothelial dysfunction has been reported in both syndromes, with changes to the microcirculation potentially causing hypoperfusion and contributing to the exercise intolerance these patients experience (Haffke et al. 2022; Scherbakov et al. 2020). Perhaps the specific combination of these underlying processes influences the varied manifestation of long COVID in individual patients. Furthermore, it has been suggested that the hyperinflammatory state seen in SARS-CoV-2 infection may work synergistically with inflammatory changes that occur secondary to the surgical stress response (Noll et al. 2022). Understanding the mechanisms causing patients’ symptoms, particularly their exercise limitation, and how these mechanisms may be affected by surgery, may help elucidate how best to manage these patients perioperatively.

Long COVID appears heterogeneous in both its presentation and underlying pathology. Pulmonary function tests (PFT) and echocardiography are frequently used tools in anaesthetic pre-assessment. Dyspnoea is one of the more commonly reported long COVID symptoms, and patients with ongoing dyspnoea up to 3 months after non-critical COVID-19 have demonstrated lower forced vital capacity, forced expiratory volume in 1 second and diffusion capacity of the lungs for carbon monoxide than those without dyspnoea (Cortés-Telles et al. 2021). They also had reduced functional capacity compared to their non-breathless counterparts, as measured by the 6-minute walk test (Cortés-Telles et al. 2021). PFTs may therefore be useful in the preoperative assessment of long COVID patients. Additionally, echocardiography may be helpful; 25% of 150 patients with long COVID symptoms, none of whom was hospitalised with COVID-19 or had pre-existing cardiovascular disease, had echocardiographic abnormalities at 4–12 weeks post-infection (Tudoran et al. 2021). Furthermore, there was a statistically significant correlation between the presence of echocardiographic abnormalities, the number of long COVID symptoms reported and a worse self-reported functional status (Tudoran et al. 2021). Conversely, studies have shown that long COVID patients with dyspnoea and fatigue can have normal PFTs and echocardiography, yet on cardiopulmonary exercise testing (CPET), there is demonstrable reduced peak aerobic capacity and impaired oxygen extraction almost a year after the initial infection (Singh et al. 2022). This suggests that perhaps our conventional pre-assessment of echocardiography and PFT could be of less value in a subset of long COVID patients, providing false reassurance of their cardiopulmonary function.

## Existing guidelines — current scope and limitations

Multiple organisations worldwide have developed guidelines for patients with prior COVID-19 infection who subsequently require elective surgery (Noll et al. 2022; El-Boghdadly et al. 2022; Cortegiani et al. 2022; American Society of Anesthesiologists and Anesthesia Patient Safety Foundation 2022; Australian and New Zealand College of Anaesthetists 2022). As summarised in Table 1, these largely focus on the suggested time interval between infection and surgery and recommendations for preoperative assessment if surgery is required before this. The expert consensus from the UK, Italy and Germany advises delaying elective procedures until at least 7 weeks post-COVID-19 infection and until symptom resolution, due to the aforementioned increased risk of postoperative mortality and pulmonary complications (COVIDSurg Collaborative, GlobalSurg Collaborative 2021; Noll et al. 2022; El-Boghdadly et al. 2022; Cortegiani et al. 2022). However, the American Society of Anaesthesiologists (ASA) only recommends this delay for unvaccinated, asymptomatic patients, stating that there is insufficient evidence for those who have unresolved symptoms or are vaccinated and subsequently infected (American Society of Anesthesiologists and Anesthesia Patient Safety Foundation 2022). Indeed, the applicability of the early pandemic data which informed the suggested time delay has been questioned; less virulent COVID strains have since attained dominance, more efficacious treatments are available and a large proportion of the population has now been vaccinated (Lieberman et al. 2022). Furthermore, given that a significantly smaller proportion of COVID patients now require hospital or critical care admission than during the initial outbreak, they argue that recommendations regarding the timing of elective surgery need updating to reflect the current therapeutic landscape (Lieberman et al. 2022). However, postoperative outcomes were not directly investigated within this study, and whilst elective surgery may indeed be safer after a shorter delay for patients who have recovered from these less virulent strains, this remains speculative. Additionally, guidance on the timing of elective surgery for long COVID patients is limited, with organisations tentatively encouraging consideration of further delay beyond 7 weeks based on a multidisciplinary risk-versus-benefit discussion (El-Boghdadly et al. 2022; Cortegiani et al. 2022). Optimal timing will therefore depend on patients’ presenting symptoms and other comorbidities, balanced against their need for surgery and the likelihood of a prolonged delay worsening their functional status or quality of life.

**Table 1:** Summary of national organisations’ guidelines and consensus statements on post-COVID-19 patients requiring elective surgery

**Organisation**	**Recommendations**		
	**Delay between COVID-19 infection and elective surgery**	**Preoperative assessment for those with recent COVID-19 infection < 7 weeks**	**Considerations for long COVID patients**
Consensus statement: Association of Anaesthetists, Centre for Perioperative Care, Federation of Surgical Specialty Associations, Royal College of Anaesthetists, Royal College of Surgeons of England (El-Boghdadly et al. 2022)	At least 10 days after infection, ideally > 7 weeks	• Assess baseline risk using validated risk assessment tools • Optimise where possible • Involve multidisciplinary decision-making, including additional assessment of patient factors, surgical factors and COVID-19-related risk	If persistent symptoms, consider further delay beyond 7 weeks based on a multidisciplinary risk/benefit assessment
Italian Society of Anesthesia Analgesia Resuscitation and Intensive Care (SIAARTI) (Cortegiani et al. 2022)	At least 10 days after infection, ideally > 7 weeks, unless the benefit outweighs the risk	• Risk assess patients using validated tools and multidisciplinary decision-making, including COVID-19-related risk and surgical factors • Optimise where possible • Use the SIAARTI preoperative assessment protocol to guide investigation requests based on surgical complexity and COVID symptoms at the time of infection	If persistent symptoms, consider further delay beyond 7 weeks based on a multidisciplinary risk/benefit assessment
Germany Society of Surgery and Germany Society of Anesthesiology and Intensive Care Medicine (Noll et al. 2022)	Ideally > 7 weeks	Nil specific	If persistent symptoms, ideally delay surgery
American Society of Anesthesiologists and Anesthesia Patient Safety Foundation (American Society of Anesthesiologists and Anesthesia Patient Safety Foundation 2022)	• 7 weeks in unvaccinated patients who are asymptomatic at the time of surgery • There is insufficient evidence to make recommendations for patients infected with COVID-19 after vaccination	Informed decision-making with the patient regarding the increased risk, taking note of illness severity, ongoing symptoms, comorbidities and surgical complexity	• Consider further delay if ongoing symptoms • These patients need a thorough preoperative assessment, focusing on the cardiopulmonary system
The Australian and New Zealand College of Anaesthetists (Australian and New Zealand College of Anaesthetists 2022)	◦ Minor surgery: 4 week delay ◦ Major surgery: delay for at least 7 weeks	• Risk assessment, considering patient, surgical and COVID factors • Shared decision-making is needed • Discuss patients with haematology regarding perioperative thromboprophylaxis	• Undertake a formal clinical review, involving all organ systems • Consider brain natriuretic peptide (BNP), ferritin and echocardiography based on the patient’s functional limitation and severity of infection • Consider a repeat chest X-ray (CXR)/CT chest • Cardiovascular or respiratory complications post-COVID-19 should be optimised
Indian Society of Anaesthesiologists (Malhotra et al. 2021)	Interval depends on the following: • Illness severity • The presence of organ damage following infection • Relevant drugs involved in the management of COVID-19	All patients should have the following: ◦ Ambulatory saturations ◦ An estimation of exercise tolerance and assessment of functional status, e.g. via a 6-minute walk test ◦ A review of affected organ systems and drug history ◦ Baseline bloods and ECG. Further investigations, e.g. CXR, echocardiography and PFTs, should depend on the patient’s ASA grade, comorbidities and the complexity of the surgery • Optimisation of their modifiable risk factors, e.g. smoking cessation	

Given the large spectrum of symptoms experienced by long COVID patients, in conjunction with a paucity of data on their postoperative outcomes, developing guidelines is complex. For those with recent COVID-19 infection, the Association of Anaesthetists consensus statement recommends assessing baseline perioperative risk using a validated risk assessment tool (e.g. Surgical Outcome Risk Tool (SORT)), optimising modifiable risk factors where possible and reaching a multidisciplinary decision based on these patient and surgical factors and considering patients’ additional COVID-19 risk (El-Boghdadly et al. 2022; Wong et al. 2020). However, these risk prediction models have not yet included long COVID in their online calculators as an independent predictor of risk. Without accurate risk prediction, having a comprehensive multidisciplinary discussion or obtaining informed patient consent is difficult. As part of their initial workup for suspected long COVID in primary care, patients should have had baseline blood tests (including full blood count, renal, liver and thyroid function tests, C-reactive protein, ferritin, D-dimer, glycated haemoglobin (HbA1c) and brain natriuretic peptide (BNP) and a resting ECG and chest x-ray (CXR) (National Institute for Health and Care Excellence 2021). Those with postural symptoms should also have had lying and standing blood pressures. An assessment of patients’ exercise tolerance, such as through a 6-minute walk test or by interrogation of their functional capacity using metabolic equivalents (METs) (Bossone et al. 2021), is required. Given the heterogeneity of the disease, it seems prudent to take a systems-based approach to further preoperative assessment. These initial steps could help identify patients that may benefit from onward specialist referral and additional preoperative investigations (such as PFTs and cardiac imaging). Functional capacity assessments such as CPET are not regularly undertaken in many NHS hospitals; perhaps referral for this could benefit those with persistent dyspnoea despite normal PFTs and echocardiography to better understand their exercise limitation and to aid quantification of their perioperative risks.

Whilst the majority of the above guidelines focus on preoperative assessment, other aspects of perioperative management for long COVID patients warrant consideration. In terms of the conduct of anaesthesia, avoiding a general anaesthetic in favour of regional or local techniques seems sensible where possible, particularly in those with severe respiratory pathology. This is also recommended for those with post-COVID-19 respiratory sequelae in consensus statements from organisations in the UK, Italy and India (El-Boghdadly et al. 2022; Cortegiani et al. 2022; Malhotra et al. 2021). Additionally, postoperative venous thromboembolism (VTE) is more common in patients with recent COVID-19, and those undergoing general anaesthesia may be at an increased risk (COVIDSurg Collaborative, GlobalSurg Collaborative 2022). Importantly, the risk of postoperative VTE was much greater in patients with ongoing COVID symptoms, even for those more than 7 weeks post-infection (COVIDSurg Collaborative, GlobalSurg Collaborative 2022). In conjunction with evidence that previous COVID-19 infection increases the risk of pulmonary embolism for up to 110 days post-infection (Katsoularis et al. 2022), it seems prudent to consider that long COVID patients may be at increased risk of VTE post-surgery. It is worth noting that, in the latter study, only 30% of the national population had had their first vaccine against COVID-19 by study completion, and Omicron had not yet become the prominent variant, so these findings may not be fully generalisable to the current immunity and virulence strains within the population (Katsoularis et al. 2022). The researchers additionally did not specifically investigate postoperative VTE or long COVID patients. Despite this, there is sufficient evidence to suggest that these patients may be at increased risk of postoperative VTE, yet prophylaxis strategies are not discussed in the majority of the above guidelines. Recommendations from the Australian and New Zealand College of Anaesthetists briefly suggest a discussion with haematology in this regard (Australian and New Zealand College of Anaesthetists 2022). It has been suggested by an expert consensus that patients up to 6 months post-COVID-19 infection should receive pharmacological thromboprophylaxis postoperatively, but how long this should be continued for has not been specified (Ferrandis et al. 2021). Finally, there is minimal guidance for determining the most appropriate postoperative destination for long COVID patients undergoing surgery. Perhaps having long COVID as a comorbidity should prompt perioperative clinicians to consider the need for high dependency level monitoring postoperatively. This should form part of a holistic assessment of other patient factors, surgical factors and the severity and nature of their long COVID symptoms.

## Conclusion

In summary, long COVID is a heterogenous and increasingly common condition, and these patients may be at increased postoperative risk. The pathophysiology of long COVID is complex; a better understanding of the underlying mechanisms may help clinicians recognise how to best optimise these patients preoperatively and predict how they will respond to the surgical stress response. Developing guidelines for the preoperative assessment of these patients is challenging due to the diverse presentation of long COVID and the lack of data on postoperative outcomes for this cohort. Timing of elective surgery will likely need to be individualised for these patients, based on their constellation and severity of symptoms, their comorbidities and the complexity and urgency of the proposed surgery; shared decision-making will be required to consider the relative importance of these factors. Perioperative clinicians should take a systems-based approach to assessing these patients preoperatively to help determine whether additional discussions with specialists and further investigations would be beneficial, in addition to their existing primary care workup. Clinicians should additionally be cautious about the potentially increased risk of postoperative VTE in these patients and consider avoiding a general anaesthetic for those with severe respiratory sequelae. Patients and clinicians require prospective studies to help to quantify these risks, enable informed consent and ensure sound perioperative management to reduce the likelihood of adverse perioperative outcomes.

## Data Availability

Not applicable

## Abbreviations

ASA: American Society of Anaesthesiologists
BNP: Brain natriuretic peptide
CPET: Cardiopulmonary exercise testing
CXR: Chest X-ray
ECG: Electrocardiogram
HbA1C: Glycated haemoglobin
ME/CFS: Myalgic encephalitis/chronic fatigue syndrome
MET: Metabolic equivalent
NHS: National Health Service
NICE: National Institute for Health and Care Excellence
PFT: Pulmonary function tests
POTS: Postural tachycardia syndrome
SIAARTI: Italian Society of Anesthesia, Analgesia, Resuscitation and Intensive Care
SORT: Surgical Outcome Risk Tool
VTE: Venous thromboembolism

## References

[CR1] American Society of Anesthesiologists, Anesthesia Patient Safety Foundation (2022). ASA and APSF Joint Statement on Elective Surgery/Procedures and Anesthesia for Patients after COVID-19 Infection.

[CR2] Ariza ME (2021). Myalgic encephalomyelitis/chronic fatigue syndrome: the human herpesviruses are back!. Biomolecules.

[CR3] Australian and New Zealand College of Anaesthetists (2022). Guideline on surgical patient safety for SARS-CoV-2 infection and vaccination.

[CR4] Bossone E, Cademartiri F, AlSergani H, Chianese S, Mehta R, Capone V (2021). Preoperative assessment and management of cardiovascular risk in patients undergoing non-cardiac surgery: implementing a systematic stepwise approach during the COVID-19 pandemic era. J Cardiovasc Dev Dis.

[CR5] Cortegiani A, Tripodi VF, Castioni CA, Esposito C, Galdieri N, Monzani R (2022). Timing of surgery and elective perioperative management of patients with previous SARS-CoV-2 infection: a SIAARTI expert consensus statement. J Anesth Anal Crit Care.

[CR6] Cortés-Telles A, López-Romero S, Figueroa-Hurtado E, Pou-Aguilar YN, Wong AW, Milne KM (2021). Pulmonary function and functional capacity in COVID-19 survivors with persistent dyspnoea. Respir Physiol Neurobiol.

[CR7] COVIDSurg Collaborative, GlobalSurg Collaborative (2021). Timing of surgery following SARS-CoV-2 infection: an international prospective cohort study. Anaesthesia.

[CR8] COVIDSurg Collaborative, GlobalSurg Collaborative (2022). SARS-CoV-2 infection and venous thromboembolism after surgery: an international prospective cohort study. Anaesthesia.

[CR9] El-Boghdadly K, Cook TM, Goodacre T, Kua J, Denmark S, McNally S (2022). Timing of elective surgery and risk assessment after SARS-CoV-2 infection: an update: a multidisciplinary consensus statement on behalf of the Association of Anaesthetists, Centre for Perioperative Care, Federation of Surgical Specialty Associations, Royal College of Anaesthetists, Royal College of Surgeons of England. Anaesthesia.

[CR10] Ferrandis R, Llau JV, Afshari A, Douketis JD, Gómez-Luque A, Samama CM (2021). Management of perioperative thromboprophylaxis for surgery following COVID-19: an expert-panel survey. Br J Anaesth.

[CR11] Haffke M, Freitag H, Rudolf G, Seifert M, Doehner W, Scherbakov N (2022). Endothelial dysfunction and altered endothelial biomarkers in patients with post-COVID-19 syndrome and chronic fatigue syndrome (ME/CFS). J Transl Med.

[CR12] Katsoularis I, Fonseca-Rodríguez O, Farrington P, Jerndal H, Lundevaller EH, Sund M (2022). Risks of deep vein thrombosis, pulmonary embolism, and bleeding after covid-19: nationwide self-controlled cases series and matched cohort study. BMJ.

[CR13] König RS, Albrich WC, Kahlert CR, Bahr LS, Löber U, Vernazza P (2021). The gut microbiome in myalgic encephalomyelitis (ME)/chronic fatigue syndrome (CFS). Front Immunol.

[CR14] Lieberman N, Racine A, Nair S, Semczuk P, Azimaraghi O, Freda J (2022). Should asymptomatic patients testing positive for SARS-CoV-2 wait for elective surgical procedures?. Br J Anaesth.

[CR15] Liu Q, Mak JWY, Su Q, Yeoh YK, Lui GCY, Ng SSS (2022). Gut microbiota dynamics in a prospective cohort of patients with post-acute COVID-19 syndrome. Gut.

[CR16] Lopez-Leon S, Wegman-Ostrosky T, Perelman C, Sepulveda R, Rebolledo PA, Cuapio A (2021). More than 50 long-term effects of COVID-19: a systematic review and meta-analysis. Sci Rep.

[CR17] Malhotra N, Bajwa SJS, Joshi M, Mehdiratta L, Hemantkumar I, Rani RA (2021). Perioperative management of post-COVID-19 surgical patients: Indian Society of Anaesthesiologists (ISA National) Advisory and Position Statement. Indian J Anaesth.

[CR18] National Institute for Health and Care Excellence (2021). Myalgic encephalomyelitis (or encephalopathy)/chronic fatigue syndrome: diagnosis and management.

[CR19] National Institute for Health and Care Excellence. COVID-19 rapid guideline: managing the long-term effects of COVID-19. 2021. Available from: https://www.nice.org.uk/guidance/ng188/resources/covid19-rapid-guideline-managing-the-longterm-effects-of-covid19-pdf-51035515742. 33555768

[CR20] Noll J, Reichert M, Dietrich M, Riedel JG, Hecker M, Padberg W (2022). When to operate after SARS-CoV-2 infection? A review on the recent consensus recommendation of the DGC/BDC and the DGAI/BDA. Langenbecks Arch Surg.

[CR21] Office For National Statistics. Prevalence of ongoing symptoms following coronavirus (COVID-19) infection in the UK: 2 February 2023. Estimates of the prevalence of self-reported long COVID and associated activity limitation, using UK Coronavirus (COVID-19) Infection Survey data. 2023. Available from: https://www.ons.gov.uk/peoplepopulationandcommunity/healthandsocialcare/conditionsanddiseases/bulletins/prevalenceofongoingsymptomsfollowingcoronaviruscovid19infectionintheuk/2february2023.

[CR22] Peluso MJ, Deveau TM, Munter SE, Ryder D, Buck A, Beck-Engeser G (2023). Chronic viral coinfections differentially affect the likelihood of developing long COVID. J Clin Invest.

[CR23] Phetsouphanh C, Darley DR, Wilson DB, Howe A, Munier CML, Patel SK (2022). Immunological dysfunction persists for 8 months following initial mild-to-moderate SARS-CoV-2 infection. Nat Immunol.

[CR24] Ruzieh M, Dziuba M, Hofmann JP, Grubb BP (2018). Surgical and dental considerations in patients with postural tachycardia syndrome. Auton Neurosci.

[CR25] Scherbakov N, Szklarski M, Hartwig J, Sotzny F, Lorenz S, Meyer A (2020). Peripheral endothelial dysfunction in myalgic encephalomyelitis/chronic fatigue syndrome. ESC Heart Fail.

[CR26] Schultheiß C, Willscher E, Paschold L, Gottschick C, Klee B, Henkes SS (2022). The IL-1β, IL-6, and TNF cytokine triad is associated with post-acute sequelae of COVID-19. Cell Rep Med.

[CR27] Singh I, Joseph P, Heerdt PM, Cullinan M, Lutchmansingh DD, Gulati M (2022). Persistent exertional intolerance after COVID-19: insights from invasive cardiopulmonary exercise testing. Chest.

[CR28] Swank Z, Senussi Y, Manickas-Hill Z, Yu XG, Li JZ, Alter G (2022). Persistent circulating severe acute respiratory syndrome coronavirus 2 spike is associated with post-acute coronavirus disease 2019 sequelae. Clin Infect Dis.

[CR29] Tudoran C, Tudoran M, Pop GN, Giurgi-Oncu C, Cut TG, Lazureanu VE (2021). Associations between the severity of the post-acute COVID-19 syndrome and echocardiographic abnormalities in previously healthy outpatients following infection with SARS-CoV-2. Biology (Basel).

[CR30] Wong DJN, Harris S, Sahni A, Bedford JR, Cortes L, Shawyer R, et al. Developing and validating subjective and objective risk-assessment measures for predicting mortality after major surgery: An international prospective cohort study. PLoS Med. 2020;17(10):e1003253. 10.1371/journal.pmed.1003253 PMC7561094 33057333

[CR31] Wynter-Blyth V, Moorthy K (2017). Prehabilitation: preparing patients for surgery. BMJ.

